# Banana pseudo-stem biochar derived from slow and fast pyrolysis process

**DOI:** 10.1016/j.heliyon.2023.e12940

**Published:** 2023-01-12

**Authors:** Nurhayati Abdullah, Rahmad Mohd Taib, Nur Syairah Mohamad Aziz, Muhammad Rabie Omar, Nurhafizah Md Disa

**Affiliations:** a Energy Studies Laboratory, School of Physics, Universiti Sains Malaysia, 11800, Minden, Penang, Malaysia; b Department of Science and Biotechnology, Faculty of Engineering and Life Sciences, Universiti Selangor, Jalan Timur Tambahan, 45600, Bestari Jaya, Selangor, Malaysia

**Keywords:** Banana pseudo-stem, Biochar, Fast pyrolysis, Slow pyrolysis, BPS, Banana pseudo-stem, FTIR, Fourier transform infrared, FESEM, Field emission scanning electron microscope, HHV, Higher heating value

## Abstract

This study evaluated the properties of banana pseudo-stem (BPS) biochar derived from two different types of pyrolysis. The fast pyrolysis experiment was performed using a worktable-scale fluidized-bed reactor, while a bench-scale fixed-bed reactor was used in the slow pyrolysis experiment. The preliminary analysis shows that the feedstock contains 80.6 db wt% of volatile matter, 12.5 db wt% of ash and 33.6% of carbon content. Biochar yield reduces as the pyrolysis temperature elevates for both pyrolysis experiments. Fast pyrolysis yields a higher percentage of biochar (40.3%) than biochar yield obtained from the slow pyrolysis experiment (34.9 wt%) at a similar temperature of 500 °C. The evaluation of biochar derived at 500 °C shows that the biochar obtained from the slow pyrolysis process has higher carbon content, heating value, and surface area with lower ash content. Meanwhile, FESEM images show significant differences in surface morphology and the number of pores for biochar derived from fast and slow pyrolysis. These findings indicate the potential and suitability of BPS biochar derived from the slow pyrolysis process in applications such as soil amelioration and solid biofuel.

## 1 Introduction

Bananas originate from South and Southeast Asia and are grown predominantly for their fruit [1]. In 2020, banana cultivation covers approximately 5.20 million hectares globally with an estimated production exceeding 119 million tonnes [2], with India, China, Indonesia, Brazil and Ecuador as the top five producers of bananas. Meanwhile, banana cultivation generated about 312,968 tonnes of total production in Malaysia, covering 21,630 ha of cultivation area [2]. In 2030, Malaysia is expected to generate more than 400,000 tonnes of bananas based on the increment trends of the total production and plantation area yearly [3]. The rise in production and the plantation area also increases the quantities of residues in the banana industry. About 4 tonnes of residues are generated for every tonne of bananas plucked [4]. Most of the wastes are generated at the plantations, which include fresh and dried leaves, pseudo-stem, and rhizomes. Their presence led to disposal problems in the plantation area, and severe ecological damages will undoubtedly happen if the agricultural waste is not managed correctly [[5], [6], [7]]. Therefore, numerous scientific studies have been performed to evaluate the potential of banana wastes such as banana peel [8,9], banana peduncle [10] and banana leaves [11] as the feedstock to produce biochar in various fields of application.

Biochar is a solid product obtained from the carbonization or pyrolysis of biomass. According to Lehmann and Joseph [12], biochar is defined as the product of the thermal decomposition of organic material in the absence or limited supply of oxygen, and at relatively low temperatures (<700 °C). Using biomass as feedstock to produce biochar has been suggested to reduce emissions from biomass that would otherwise naturally decay into greenhouse gases. The primary application of biochar is as a soil amendment to enhance soil functions. Applying biochar to soil also has been described as a way of sequestering atmospheric carbon dioxide (CO_2_) [12,13]. Previous studies also demonstrated that the application of biochar into soil resulted in improved soil fertility [14,15]. The potential of biochar in other applications, such as solid fuel and adsorbents also have been reported in previous works [8,[16], [17], [18], [19]].

The assessment of biochar properties is essential in biochar research to meet the specific needs of the related applications. Abdullah and Wu [20] found that the biochar derived from mallee wood exhibits desirable fuel properties, with lower moisture, lower ash, and lower sulphur and nitrogen contents. The high ash content of biochar may inhibit its potential in fuel application as higher ash content in fuel leads to higher dust emission and air pollution, besides affecting combustion efficiency [21]. Meanwhile, a high percentage of fixed carbon content in biochar is usually desirable in fuel as it allows the fuel to release more heat energy during combustion besides having the capability to burn longer [22]. On the other hand, other properties such as pH values, specific surface area (SSA) and porosity should be considered for biochar application as soil enhancers. Biochar with high organic carbon content, high SSA, and more porous structures will increase the adsorption capacity [23], hence increasing its function as an enhancer. Also, it is essential to note that all biochar has different properties. The characteristics of biochar greatly vary according to the nature of the raw feedstock used and the pyrolysis process conditions.

Pyrolysis is a thermochemical decomposition process that applies to any carbon-based product [24,25]. Pyrolysis offers good opportunities from the sustainable development point of view. It allows the use of a wide variety of materials as feedstock. Also, it produces fewer greenhouse gas emissions than other incineration technologies [[25], [26], [27]]. Nevertheless, CO_2_ emissions from the pyrolysis of biomass can be treated as net zero by assuming that the CO_2_ released is captured during the biomass growth cycle. Biomass pyrolysis also can be carbon negative if its product, such as biochar, is buried underground for carbon credits and crop enhancement [28].

The operating variables, such as pyrolysis temperature, heating rate and residence time are the main parameters in determining the types and percentages of products derived from the pyrolysis process. According to Brown et al. [29], the main differences between slow and fast pyrolysis are the heating rates and maximum reaction temperatures. Slow pyrolysis heating rates are usually around 5 - 20 °C/min, while fast pyrolysis can achieve rates exceeding 1000 °C/min. Slow pyrolysis requires several minutes or even hours, while fast pyrolysis can be performed in a short time as little as 2 s. The gap in time results in significant differences in the product distributions.

Additionally, during the slow pyrolysis process, the reactions taking place are always in equilibrium [30]. The heating duration is sufficiently slow to allow equilibration during the pyrolysis process. In this case, the heating rate limits the ultimate yield and product distribution. Hence, the products' residence time in the reactor becomes another important factor since their presence can influence both primary and secondary reactions. Conversely, there is a negligible number of reactions during the heat-up period of fast pyrolysis. Whatever pyrolysis reactions occur take place isothermally at the terminal temperature. In all cases of pyrolysis, the decomposition process occurs at a moderate temperature in which the biomass is heated without oxygen or air to produce a mixture of condensable liquids, gases, and char [[31], [32], [33], [34], [35]].

Pyrolysis parameters also play significant roles in determining the characteristics of pyrolysis products. Pyrolysis temperature is the main factor in determining the properties of biochar [36,37]. Higher pyrolysis temperature leads to a decrement in biochar yield. Similar results were reported in many other studies using different biomass feedstocks such as oil palm trunk [38], oil palm empty fruit bunch [39,40], oil palm shell [41] and oil palm pressed fruit fibre [42], tapioca wastes [37], corn stover [43], pine [44], almond shell and nutshell [45]. Conversely, higher pyrolysis temperatures produced biochar with higher carbon content, lower volatile matter and oxygen composition, and higher ash content [46,47]. Studies related to advanced pyrolysis techniques have been developed in the literature to enhance the existing pyrolysis process by reducing secondary reactions, lowering the reaction temperature, increasing the yield and quality of products, and allowing a more selective heating mechanism. These include microwave pyrolysis, solar pyrolysis, vacuum pyrolysis, catalytic pyrolysis, co-pyrolysis and carbon dioxide pyrolysis [48,49].

The slow pyrolysis process is usually performed to produce and characterize the properties of biochar, which is the main product of slow pyrolysis experiments. Lam et al. [8] used banana peel and orange peel in the slow pyrolysis experiment at a heating rate of 10 °C/min to produce biochar as an adsorbent in the treatment of palm oil mill effluent. The potential of biochar from slow pyrolysis experiments in multi-application, such as biofuel and soil enhancers, also have been reported in previous studies [9,13,47,50]. On the other hand, the fast pyrolysis process is a promising way of recovering bio-oil products. In previous studies, fast pyrolysis experiments are usually conducted to obtain and evaluate the liquid yield as the potential biofuel [32,51,52]. However, there are also studies reported on the characterization of biochar derived via the fast pyrolysis process. Wang et al. [53] evaluated the properties of rice husk and elm sawdust biochar and the effects of biochar application on soil quality. In another work by Mazlan et al. [54], a study of biochar characterization derived via fast pyrolysis meranti wood sawdust was done.

There are limited studies in the literature that report the comparison of fast and slow pyrolysis of biomass samples. Duman et al. [55] conducted slow and fast pyrolysis of cherry seeds and cherry shells in fixed-bed and fluidized bed reactors at different pyrolysis temperatures to evaluate the properties of the pyrolysis products. It was summarized that both temperature and reactor type influence the properties of chars and bio-oil. The findings of the study indicated that all chars obtained are suitable for household briquette production due to low ash and sulphur content, <3.6 wt% and <0.2 wt% respectively. In another study by Al Arni [56], slow and fast pyrolysis of sugarcane bagasse was performed, and they evaluated the comparison of syngas derived from both types of pyrolysis. The results show that fast pyrolysis produced more bio-oil than slow pyrolysis. Concurrently, slow pyrolysis resulted in a higher biochar and syngas yield than the fast pyrolysis process. The properties of biochar and bio-oil were not reported in this study. A recent work by Adelawon et al. [57] studied the comparison of slow, fast and flash pyrolysis of maize-cob waste at a similar terminal temperature, 600 °C. The results show that slow pyrolysis produced the highest biochar yield, 38.40% while the highest bio-oil yield (46.25%) was obtained from flash pyrolysis followed by fast pyrolysis which yields 43.13% of bio-oil. To the best of the author’s knowledge, no research work was reported on the comparison of fast and slow pyrolysis of banana wastes. Additionally, there has yet to be any study reported on the comparison of biochar produced from a similar type of banana waste via different types of pyrolysis.

**Fig. 1:**
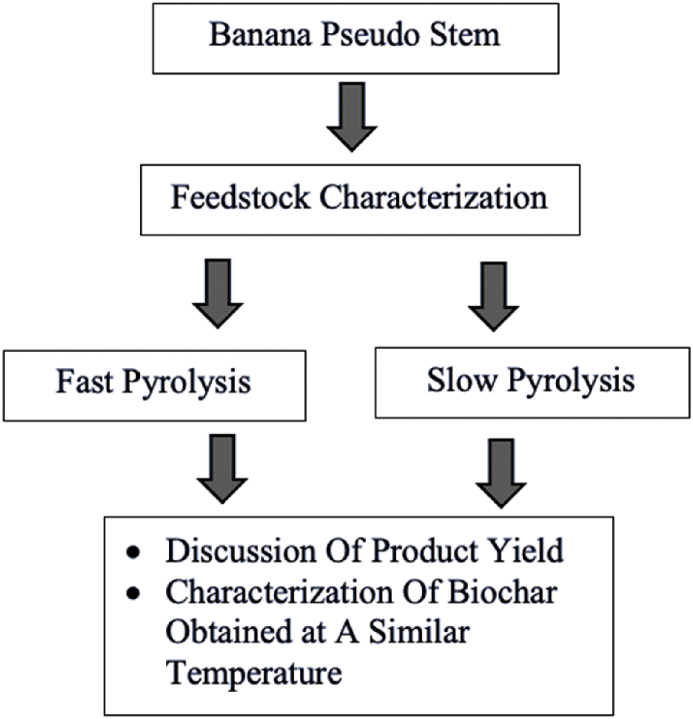
Logic diagram.

Hence, this work is conducted to evaluate the properties of banana pseudo-stem (BPS) biochar derived from fast and slow pyrolysis. This study also focuses on evaluating and explaining the potential of banana pseudo-stem as a viable and practicable feedstock for different types of pyrolysis processes. The yield and composition of products obtained from the fast pyrolysis using the fluidized-bed reactor were compared with those obtained from a fixed-bed reactor in the slow pyrolysis experiment. The characteristics of BPS biochar are also investigated to evaluate its potential in various applications, such as soil amendment and solid biofuels. The finding of this study will contribute to a better understanding of the influence of pyrolysis conditions on the biochar properties derived from banana waste. A logic diagram of this research is presented in Fig. 1.

## 2 Materials and methods

### 2.1 Collection and preparation of feedstock

BPS feedstock used in this study was obtained from the banana plantation at Sik, the largest district in Kedah, Malaysia. Its geographical coordinates are 5^◦^ 49’ 0” North, and 100^◦^ 44’ 0” East. The BPS feedstock was derived from the type of Musa acuminata cv. Emas. This type of banana is used in this study due to the highest demand for distribution in Malaysia compared to other cultivars. Only matured banana trees, which are typically more than 11 months aged were chosen and used in this study. They were cut around 25 cm off the ground. Then, the samples were chopped into smaller sizes (5–10 pieces) to ease the handling and transportation of the sample.

**Fig. 2:**
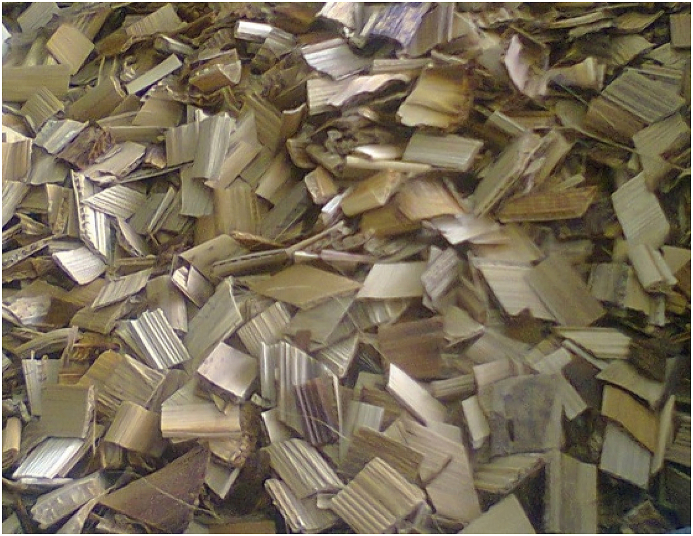
Dried banana pseudo-stem.

As the sample was in wet condition during the collection, the BPS was dried in the oven at 105 °C (Venticell 222 Standard) until its moisture content was less than 10 db wt% to avoid the growth of fungus and microorganisms. The sample was cut into pieces of 4 cm × 4 cm before drying, as shown in Fig. 2.

### 2.2 Size reduction and feeding test for fast pyrolysis

In fast pyrolysis, the chemical reaction takes place quickly (within 2–2.5 s). Therefore, a feedstock with tiny particle sizes (finely ground) is required to allow a higher heat transfer rate and consequently meet the conditions of fast heating to a uniform temperature [58]. A Retsch cross-beater mill with a screen size of 1000 μm was used to reduce the sizes of the samples. Meanwhile, slow pyrolysis does not require finely ground feedstock, considering that the process occurs for a longer time and a high rate of particle heating is unnecessary.

A feeding test was performed before the fast pyrolysis experiment to determine the suitable particle size for the experimental procedure. It is desirable to have a particle size distribution as narrow as possible. The feedstock was grouped into five different size ranges, as reported, and presented in the previous study [3]. A 300 g/h reactor is used in the fast pyrolysis experiment. Therefore, this study also included a feeding test for this reactor. Table 1 displays the distribution percentage and the feed rate for various particle size ranges.

**Table 1:** Mass distribution and feed rate of BPS.

Feed particle size (μm)	Distribution (%)	Feed rate (g/h)
800–1000	3	103.3
600–800	19	132.5
400–600	18	189.4
224–400	24	81.8
0–224	36	0

The outcome from the feeding test in Table 1 indicates that most of the particles were smaller than 224 μm. However, the feed rate of the sample indicated that only particles larger than 224 μm are acceptable to feed in the reactor for the fast pyrolysis process. Thus, the sample with the size range of 400–600 μm was selected as it provides the optimum feeding rate for the fast pyrolysis experiment. Additionally, selecting the feedstock with the smallest particle size is avoided to prevent it from overheating or being too quickly blown from the reactor before pyrolysis takes place producing more char. Also, larger particles may not be adequately heated up so causing incomplete pyrolysis as reported by Scott and Piskorz [59].

### 2.3 Feedstock characterization

The properties of BPS were evaluated via proximate, elemental, lignocellulosic, heating value and thermogravimetric analysis. The basic functional groups of BPS feedstock were analyzed by Fourier Transform Infrared (FTIR) spectroscopy. The details of these analyses were presented in the previous work [3]. Additionally, the surface morphology of the feedstock was examined via Field Emission Scanning Electron Microscope (FESEM). The analysis was performed using FEI Nova NanoSEM450.

### 2.4 Experimental procedure

The experimental procedure wasdivided into two parts: slow pyrolysis and fast pyrolysis. The details of each pyrolysis experiment are presented in the following section. The slow and fast pyrolysis experiments were replicated three times and the average value of products yield was presented as the result of this study. The standard deviation of the data is provided in the graph of products yield.

#### 2.4.1 Slow pyrolysis

**Fig. 3:**
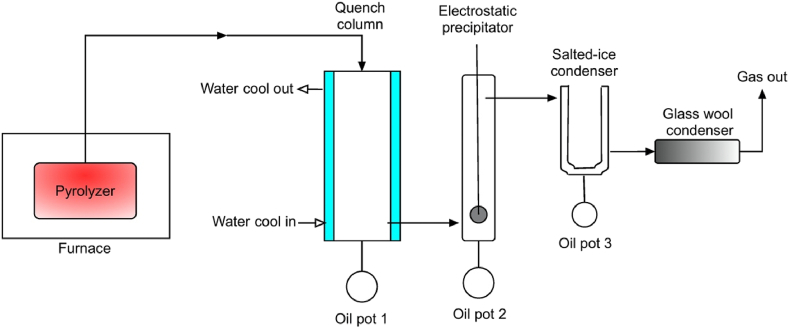
Schematic diagram of slow pyrolysis.

A slow pyrolysis experiment was performed using a bench-scale fixed bed reactor. Fig. 3 shows the schematic diagram of the slow pyrolysis setup.

**Fig. 4:**
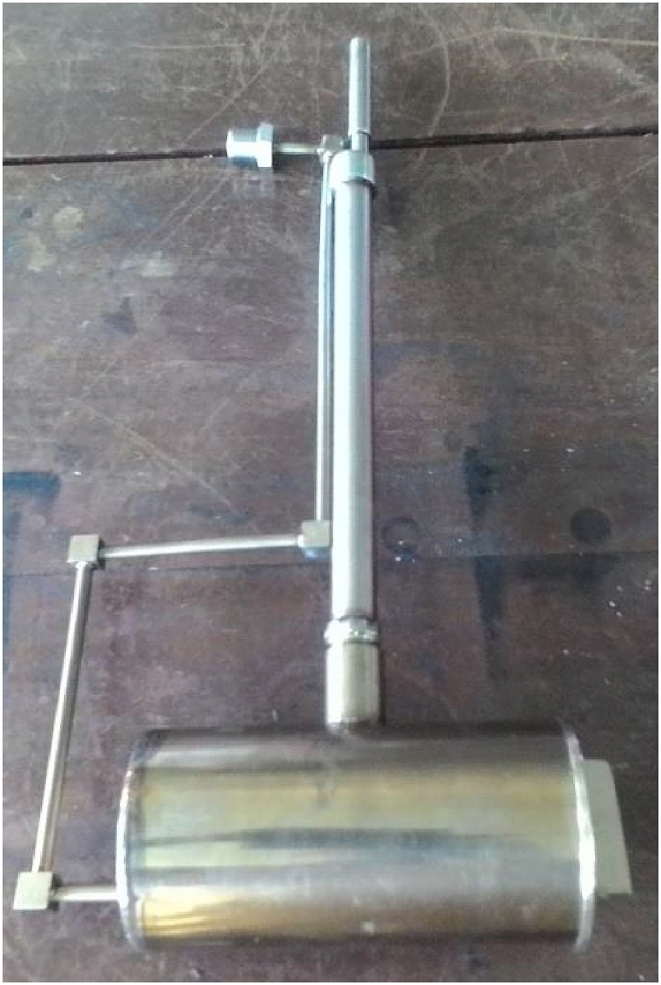
Fixed bed pyrolyzer to place the feedstock.

The slow pyrolysis setup consists of a fixed bed pyrolyzer (Fig. 4) with 70 mm internal diameter and 150 mm in length inside a furnace and a liquid collection scheme. The BPS was compacted into the pyrolyzer and then placed in the furnace. A K-type thermocouple measures the temperature inside the pyrolyzer. The experiment was performed under a nitrogen atmosphere with a constant flow rate of 0.5 L/min. The temperature was set at 400 °C. The heating rate was fixed at 10 °C/min. After a specified holding time was completed, the pyrolyzer was removed from the furnace, and let to cool down for 2 h. The experiment was repeated with the temperature of 450 °C, 500 °C, 550 °C and 600 °C. The percentage of biochar yield was determined as follows:

(1) Biochar yield, wt % = ((mass of biochar (g) / mass of dry feedstock (g)) x 100

Meanwhile, the percentage of liquid and gas yield were determined as follows:

(2) Liquid yield, wt % = ((mass of liquid (g) / mass of dry feedstock (g)) x 100

(3) Gas yield, wt% = 100 – biochar yield (%) – liquid yield (%)

These three parameters defined in equations (1, 2, 3) allow us to assess the pyrolysis process performance for a given feedstock. From here, deductions can be made whether the given pyrolysis process setting is optimum. Briefly, the biochar yield is given by the percentage ratio between the mass of biochar and the mass of dry feedstock. Next, the liquid yield is simply the percentage ratio between the mass of the liquid product and the mass of dry feedstock. Of course, the gas yield is the remaining yield fraction, as stated in equation (3).

#### 2.4.2 Fast pyrolysis

**Fig. 5:**
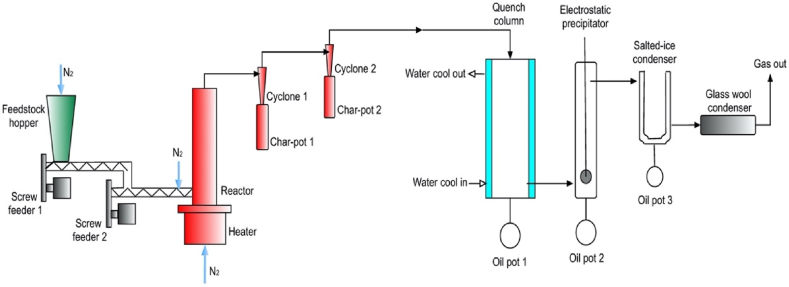
Schematic diagram of fast pyrolysis.

The fast pyrolysis experiment was carried out using a fluidized bed reactor with a 300 g/h worktable-scale capacity. The schematic diagram of the fast pyrolysis setup is presented in Fig. 5. Further details of the fast pyrolysis experimental setup have been presented in the previously published work [3], which assessed and reported the production of bio-oil via fast pyrolysis process.

The fast pyrolysis experiment of BPS feedstock was performed at various temperatures; 470, 480, 490, 500, 510, 520, 530 and 540 °C. Meanwhile, the vapour residence time was fixed at 1.02 s. The heating medium in the reactor is inert sand was sizes ranging from 400 μm to 600 μm so that the fluidising velocity of the sand was sufficient to blow the pyrolysed biomass [char and vapours] completely off the bed while the sands remain in the bed. The percentage of product yield were calculated using equations (1), (2), (3).

### 2.5 Analysis of biochar

The analysis of biochar derived from both fast pyrolysis and slow pyrolysis was performed to evaluate the properties of BPS biochar. Proximate analysis was carried out to determine the moisture content, volatile matter and ash content of BPS biochar according to ASTM 1762 [60]. The fixed carbon is the difference between 100 and the sum of ash content and volatile matter. The proximate analysis is expressed on a dry basis (db wt%). The percentage of carbon, hydrogen, nitrogen and sulphur contents of the biochar was determined using PerkinElmer Series II CHNS/O 2400 Elemental Analyser. The oxygen content was calculated through the difference of the sum of the others in relation to the total sample.

A bomb calorimeter (IKA C-200) was used to determine the higher heating value (HHV) of the BPS biochar. A sample with a known mass of around 0.5–0.7 g was placed in the bomb. The oxygen gas was fed into the bomb reaching up to 30 Barr. The bomb was then placed in the inner cylinder cautiously. The analysis was performed in triplicate. Meanwhile, the pH value of BPS biochar was evaluated following ASTM D3838-80 [61], where the solution of bio-char had to be prepared before measurement. The ground BPS char was mixed with distilled water with a ratio of 1:10. The pH was measured using a Hanna pH analyser. The test was repeated three times and the average pH value was calculated.

The surface morphology of biochar was examined via FESEM analysis. This analysis was conducted using FEI Nova NanoSEM450. The surface area and pore volume of BPS biochar were determined using Brunauer, Emmett and Teller (BET) analysis. This analysis was carried out using iDB micrometrics ASAP2020.

This study also analyzes the basic functional groups of BPS biochar using FTIR spectroscopy (Thermo Scientific Nicolet iS10). The procedure for this analysis is identical to the method performed to obtain IR spectra of the BPS feedstock which was reported in the previous work [3].

## 3 Results and discussion

### 3.1 Feedstock characterization

The properties of BPS feedstock are presented in Table 2.

**Table 2:** Characteristics of BPS feedstock.

Analysis	Property	Result
Proximate analysis (db wt.%)	Moisture	10.2
	Volatile matter	80.6
	Ash	12.5
	Fixed carbon	6.9
Elemental analysis (%)	C	33.6
	H	7.3
	N	22.1
	S	0.2
	O	36.9
Lignocellulosic analysis (wt.%)	Cellulose	32.5
	Hemicellulose	13.0
	Lignin	32.2
	Extractives	9.8
Heating value (MJ/kg)	HHV	12.4

The results of the proximate analysis of BPS feedstock are presented in dry basis percentage (db wt.%). It showed that BPS feedstock contained 80.6 db wt% of volatile matter, 12.5 db wt% of ash content and 6.9 db wt% of fixed carbon. The volatile matter of BPS feedstock used in this study is high and comparable to other non-woody feedstock used in the pyrolysis experiments, such as sunflower seed hulls [62] and corn cob [63]. The high percentage of volatile matter in the feedstock composition indicates high susceptibility to thermal degradation [64]. The ash content of BPS feedstock is 12.5 db wt%. It is higher than the result reported by Kabenge et al. [64] for BPS and also other banana residues like banana peels and leaves. Such variation could be due to a few factors such as geographic location and conditions during sample collection.

The results from the elemental analysis show that carbon and oxygen are the main components of BPS feedstock composition. However, the carbon concentration in BPS feedstock is lower than other non-woody biomass like maize residues [65] and oil palm empty fruit bunch [66,67]. The elemental analysis also revealed that the BPS feedstock used in this work contains high nitrogen content compared to other banana residues reported in the published works [64]. Such an observation could be due to the utilization of mineral-based fertilizers like phosphorus and nitrogen fertilizers instead of organic fertilizers, which consequently influenced the composition of BPS. Conversely, BPS feedstock only has 0.2 db wt% of sulphur. The low percentage of sulphur in biomass leads to a lower emission of sulphur oxides (SO_x_) during the combustion or thermochemical conversion process.

BPS feedstock contains 32.5% of cellulose, 13.0% of hemicellulose and 32.2% of lignin. In contrast to the values reported by the other literature [64], the BPS feedstock used in this study has a higher percentage of lignin which will lead to higher char formation [68]. The high heating value (HHV) of the BPS feedstock is 12.4 MJ/kg. It is almost similar to the HHV of BPS reported by Leiter et al. [69]. Nevertheless, the HHV of BPS feedstock in this research is less than the biomass feedstock used in the other pyrolysis experiments, such as oil palm kernel shell (19.5 MJ/kg) [70], cassava rhizome (20.3 MJ/kg) [71] and, maize cobs (18.23 MJ/kg) [65]. The low HHV of BPS feedstock is associated with the low percentage of fixed carbon and high ash content, as shown by the proximate analysis. A higher ash composition in biomass will reduce its calorific value. Also, a greater fixed carbon content promotes the generation of heat during combustion, which results in a higher calorific value [72].

**Fig. 6:**
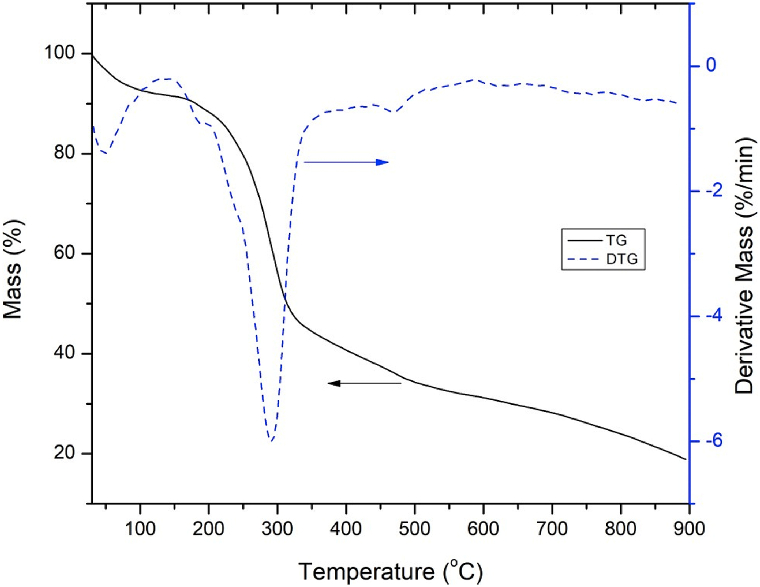
TG and DTG curves of BPS feedstock.

The thermal degradation profile of BPS feedstock is presented by TG and DTG curves as shown in Fig. 6.

From the TG analysis, the degradation of BPS feedstock occurs in a few different phases. In the initial phase, dehydration or moisture removal of BPS feedstock occur before the temperature achieved 200 °C. It is followed by the main degradation reaction, which occurs at the temperature range of 170–560 °C.

The weight loss of feedstock at a lower temperature (170–300 °C) is associated with the degradation of hemicellulose. At this range of temperature, the initial stages of cellulose degradation also occur, while the later stages of cellulose degradation occur at a higher temperature (300–360 °C). Thermal breakdown of lignin components occurs at temperatures ranging from 170 to 560 °C.

The DTG curve shows that the degradation of BPS feedstock is significant between 214 and 330 °C. A distinct peak around 289 °C indicates the maximum thermal decomposition rate of lignocellulosic elements of BPS feedstock, such as hemicellulose and cellulose. Also, the decomposition rate was 6.006%/min.

The data from TG and DTG curves of TG analysis are advantageous to determine the suitable temperature for the pyrolysis experiment of the BPS feedstock. The DTG curve in Fig. 6 shows that no significant peaks are after 400 °C, which implies that the main degradation of BPS feedstock is not occurring beyond this temperature. Additionally, the TG curve also indicates that the weight of CF decreases gradually after achieving a temperature of 400 °C. These findings demonstrate that 400 °C is the minimum pyrolysis temperature of BPS feedstock.

**Fig. 7:**
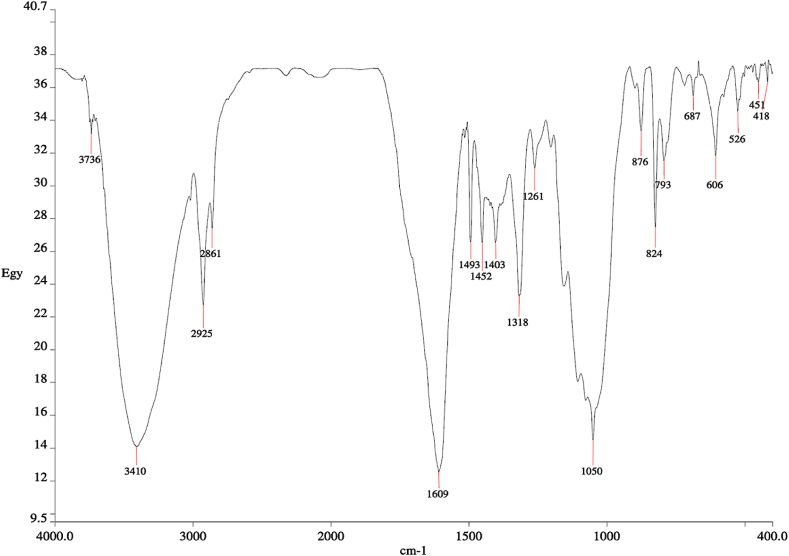
FTIR spectrum of BPS feedstock.

Fig. 7 displays the absorption frequency spectrum and the details of corresponding functional groups of BPS feedstock are summarized in Table 3.

**Table 3:** FTIR functional group of BPS feedstock.

Wave number range (cm^−1^)	Wave number (cm^−1^)	Details
3200–3600	3410, 3425, 3736	O–H bond of lignocellulosic components
3100–3500	3410, 3425	N–H stretching
2850–3000	2861, 2923, 2925	C–H stretching vibration in hemicellulose composition [73]
1400–1600	1403–1494	C=C stretching, associated with the aromatic skeletal mode of lignin
1210–1320	1261, 1318, 1320	C–O stretching of alcohols and carboxylic acids in the hemicellulose structure [73]
1300–950	1261, 1050	C–O stretching
		O–H bending

**Fig. 8:**
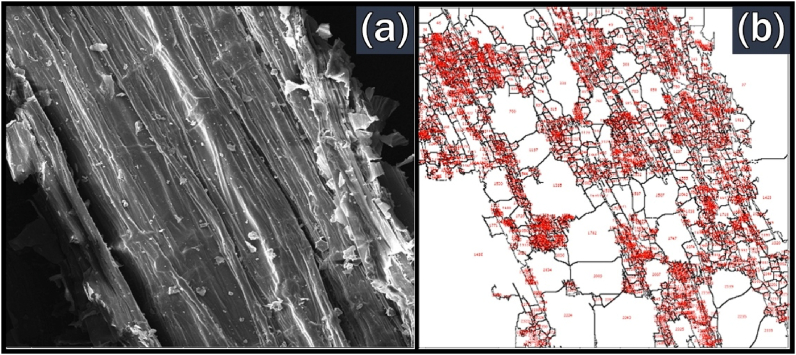
Images of raw BPS from (a) FESEM; (b) ImageJ analysing no. of pores.

The image of raw BPS from the FESEM analysis is shown in Fig. 8(a).

The FESEM image shows that BPS feedstock has a defined cellular structure with the matter remaining intact. The horizontal lines on the surface indicate the presence of bundle structure of the fibres. According to Subagyo and Chafidz [74], each of these bundles consists of several fibres. Meanwhile, Fig. 8(b) shows the corresponding image generated using the ImageJ program with the pore identification numbers. It shows that the number of pores for BPS feedstock is 2419.

### 3.2 Product yield

The products of the pyrolysis experiments are solid biochar, liquid and gas. The details of product yield obtained from slow and fast pyrolysis are presented in next following sections.

#### 3.2.1 Slow pyrolysis

**Fig. 9:**
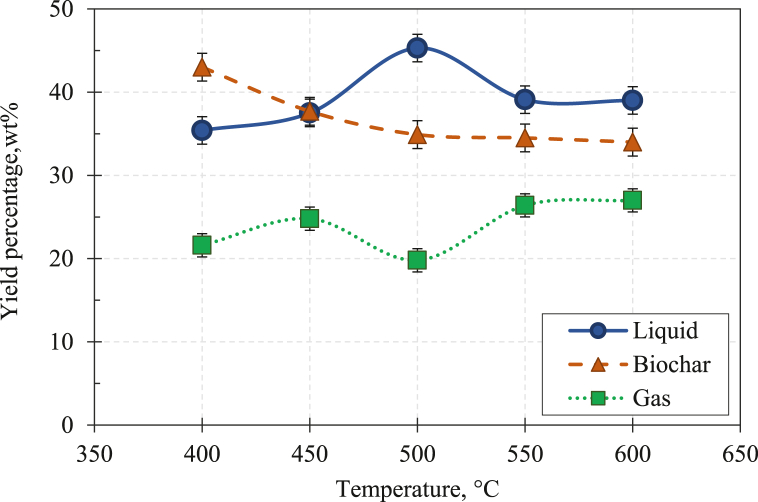
Products yield as a function of temperature for slow pyrolysis of BPS.

The percentage yield of slow pyrolysis products at various pyrolysis temperatures is presented in Fig. 9.

The results in Fig. 9 show that the percentage of biochar decreases as the pyrolysis temperature rises from 400 to 600 °C. Additionally, the decrement of BPS biochar yield is notable between 400 and 500 °C, where the yield plummets from 43.0 wt% to 34.9 wt%. Then, it gradually decreases to 34.0 wt% when the slow pyrolysis temperature reaches 600 °C. The reduction of biochar yield with increasing pyrolysis temperature is associated with the more significant primary decomposition of the biomass at high temperatures and secondary thermal decomposition of the char formed before being entrained out of the reaction zone [71]. Similar observations have been reported in other slow pyrolysis experiments in the published works [39,75,76].

Additionally, the observation in Fig. 9 also indicates that at a temperature lower than 450 °C, slow pyrolysis of BPS produced a higher biochar yield than other pyrolysis products. Over this temperature, a substantial volatile release occurred due to the thermal cracking of feedstock compositions. Such a phenomenon explains the higher percentage of liquid yield than the biochar yield beyond 450 °C in a slow pyrolysis experiment.

Meanwhile, it can be observed that the yield percentage of liquid BPS is optimum at 500 °C. At such a pyrolysis temperature, the obtained liquid yield was 45.3 wt%. The result shows the liquid yield increased from 29.8 to 45.3 wt%, with the increasing pyrolysis temperature from 300 to 500 °C. Then, the liquid yield decreased from 45.3 to 37.5 wt% as the temperature elevated up to 700 °C. Besides the reduction of liquid yield, the gas yield increased at higher pyrolysis temperatures. It is associated with the secondary cracking reactions of the pyrolysis vapours which occur at higher temperatures. The gas yield increase at higher temperatures is also due to the secondary decomposition of the char which generates non-condensable gaseous products [77].

#### 3.2.2 Fast pyrolysis

**Fig. 10:**
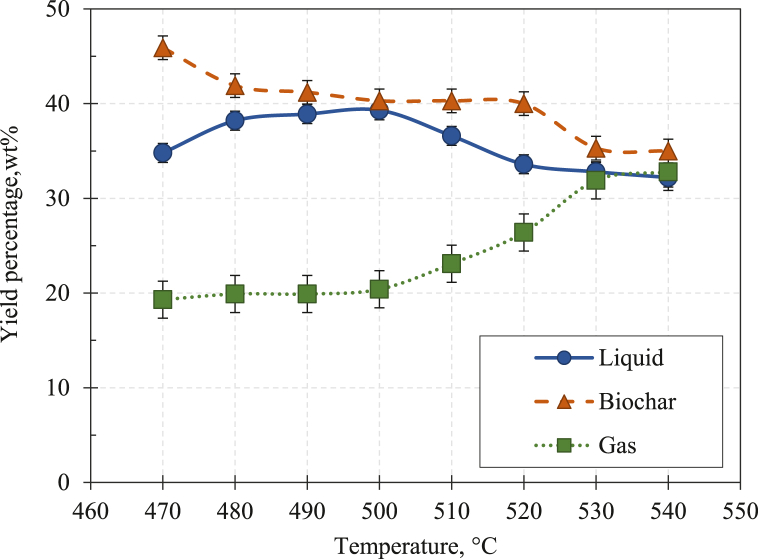
Products yield as a function of temperature for fast pyrolysis of BPS.

The percentage yield of fast pyrolysis products at various pyrolysis temperatures is presented in Fig. 10.

Fig. 10 shows the percentage of product yield from fast pyrolysis of BPS at different temperatures. The results depict that the fast pyrolysis temperature significantly influences the distribution of the products despite the small range of temperature compared to the slow pyrolysis process.

Biochar yield decreased gradually from 45.9 wt% to 32.8 wt% when the pyrolysis temperature elevated from 470 °C to 540 °C. This observation is due to the more significant primary degradation of the feedstock at higher temperatures and secondary thermal degradation of the char produced before being entrained out of the reaction zone [71]. Besides, the biochar yield obtained from the fast pyrolysis experiment at 500 °C is 40.3 wt%. A lower biochar yield was obtained at a similar temperature in the slow pyrolysis experiment, about 34.9 wt%. A similar observation was also reported in the previous study of slow and fast pyrolysis of rapeseed by Onay and Kockar [30]. Such an occurrence might be influenced by the product residence time in the reactor. It is another crucial factor that affects the yield of pyrolysis products in biomass samples. The lower biochar yield in the slow pyrolysis of BPS is possibly due to the longer residence time experienced by the char itself in the reactor, where the secondary thermal degradation of the char itself took place.

Like the findings in the slow pyrolysis experiment, the highest liquid yield was obtained at 500 °C. The liquid yield increased from 34.8 to 39.3 wt% as the temperature elevated from 470 °C to 500 °C. Subsequently, it decreased from 39.3 to 32.8 wt% when the fast pyrolysis temperature elevated to 540 °C. The decrement of liquid yield can be explained by the occurrence of secondary cracking of pyrolysis vapour which then increased gas yield. From Fig. 9 it can be observed that the percentage of gas yield increased significantly after 500 °C.

Product yield as a function of temperature for fast pyrolysis of BPS, as shown in Fig. 10 is similar to the values reported by Hauge [78] and Toft [79]. Hauge found that the maximum organic liquid yield is obtained at 400–600 °C, depending on the feedstock type. Meanwhile, Toft found that woody feedstocks, which have low ash content, give the highest organic yields and non-woody feedstocks such as rape meal and miscanthus, which have high ash content, give the lowest liquid yield. In comparison to earlier study [31], the maximum liquid yield achieved in this research is lower. It is due to the characteristics of the biomass used in this work, which has a considerably high ash concentration of 12.5 db wt%. The presence of ash in the feedstock sample has a profound effect on the pyrolysis product and liquid yields. High ash content in biomass generally promotes secondary reactions of primary pyrolysis products. Subsequently, it produces more char and gas and fewer organics. Such a phenomenon also explains the higher biochar yield than the liquid yield obtained from the fast pyrolysis of BPS in this study.

### 3.3 Properties of BPS biochar

Table 4 shows the evaluated properties of BPS biochar derived from the slow pyrolysis and fast pyrolysis of BPS feedstock. The biochar produced at 500 °C is selected for evaluation and comparison as 500 °C is the common temperature for both pyrolysis experiments in this study.

**Table 4:** Properties of BPS biochar produced at 500 °C.

Analysis	Fast Pyrolysis	Slow Pyrolysis
*Proximate analysis (db wt%)*		
Moisture content	11.4	6.1
Volatile matter	28.5	33.2
Ash content	39.3	34.0
Fixed carbon	32.2	32.8
*Elemental analysis (%)*		
C	41.28	43.23
H	0.09	0.00
N	28.48	19.96
S	0.00	0.00
O	30.15	36.81
HHV (MJ/kg)	13.6	23.4
pH	10.11	10.16
*BET*		
Surface area (m^2^/g)	0.638	1.078
Pore volume (cm^3^/g)	0.004	0.005

Results in Table 4 show notable differences in the BPS biochar properties derived from the fast and slow pyrolysis experiments. The BPS biochar produced at 500 °C by slow pyrolysis has a higher percentage of volatile matter. The higher volatile matter in the biochar composition is due to the low rate of volatile release during pyrolysis. Such a slow volatile release is the result of a lower heating rate in a slow pyrolysis experiment. With the low heating rate in the pyrolysis experiment, weak chemical bonds of the feedstock break, and these ruptures affect the structure of polymers. Such a phenomenon enables the rearrangement reactions to form a more stable matrix and inhibits the formation of volatile compounds [80]. The volatile matter in biochar composition influenced the stability of biochar, sorption capacity and plant growth in the soil application [81].

Ash content of biochar represents the non-combustible and non-volatile matter component in the biochar composition. The result from the proximate analysis showed that slow pyrolysis BPS biochar also has lower ash content, 34.0 db wt%, compared to 39.3 db wt% of ash content from the fast pyrolysis experiment. This result is in accordance with the findings reported by Angın [82]. Low ash content in biochar composition is preferable for solid fuel applications as ash content is inversely proportional to heating value.

The elemental analysis showed that carbon and oxygen are the main components of BPS biochar. Slow pyrolysis BPS biochar has a slightly higher carbon content, 43.23%, which agrees with the result of fixed carbon in proximate analysis. Meanwhile, the percentage of hydrogen in BPS biochars are very low due to the loss of hydrogen component during the pyrolysis process of BPS feedstock. The feedstock loses surface functional –OH groups due to dehydration at lower pyrolysis temperatures and loses H atoms due to the structural core degradation [83]. The result also revealed that BPS biochar contained 28.48% and 19.96% of nitrogen for biochar derived from fast and slow pyrolysis, respectively. These values are higher than the percentage of nitrogen of biochar derived from other biomass such as paddy straw, sugarcane bagasse [84], coconut shell, acacia wood [83] and apple tree branch [85], produced at a similar temperature. It could be due to the amount of nitrogen in the BPS feedstock being higher than the other type of feedstock in the literature. BPS biochars derived from both types of pyrolysis may be suitable for plant growth. Here, these biochars are rich in nitrogen, which is one of the essential plant nutrients [86] and it can increase the retention and uptake of nitrogen into the plants [87].

Heating value is one of the biochar properties that can be used as an indicator of the potential of biochar as fuel. The determination of the heating value of BPS biochar reveals that the HHV of biochars derived from the fast pyrolysis experiment (13.6 MJ/kg) is lower than the one derived from the slow pyrolysis process (23.4 MJ/kg). The distinction of HHV for biochars derived from both types of pyrolysis is associated with the percentage of carbon, ash content and moisture content that existed in the biochar. It could be observed that the heating value of biochar is directly proportional to the percentage of carbon and inversely proportional to the amount of ash and moisture content. Additionally, the result off HHV analysis also indicates that BPS biochar derived from slow pyrolysis experiment has a better quality as potential solid fuel because the heating value is similar to lumpy coal (23–25 MJ/kg), which is usually used in the boilers as reported by Manyuchi et al. [88].

For the application of biochar into the soil, the determination of biochar pH is essential as it will affect the pH of the soil. The pH of the soil is one of the important properties which influences the availability of nutrients absorption. The pH of both BPS biochars derived from fast and slow pyrolysis is alkaline, 10.11 and 10.16 respectively. The alkalinity of biochar is usually associated with the contribution of inorganic alkalis (such as Na and K) as well as the formation of carbonate (such as CaCO_3_ and MgCO_3_) which become significant around 500 °C [84,89]. The alkaline property of BPS biochar will help in neutralizing the acidic soils which resulted from the long-term application of nitrogen compounds fertilizer, hence improving the soil quality and increasing crop productivity [84].

Surface area and pore volume are vital properties for biochar as a soil enhancer. It determines the capability of water and nutrients adsorption. Results of BET analysis in Table 4 show that BPS biochar from the fast pyrolysis has a surface area of 0.638 m^2^/g, while the slow pyrolysis experiment produced biochar with a higher surface area, 1.078 m^2^/g. Biochar obtained at high heating rates has a lower surface area due to rapid depolymerization at the surface of the biochar [90]. However, an insignificant difference was observed in the pore volume of BPS biochar produced from both pyrolysis experiments. The surface area of BPS biochar found in this study was also lower compared to the biochar produced from other biomass at similar pyrolysis temperatures as reported by Lee et al. [84] and Chatterjee et al. [91]. This is due to the high percentage of ash contained in the BPS biochar compared to the biochars derived from other biomass reported in the works of literature which have lower ash content. High ash content resulted in low surface area as ash leads to pore blockage [92].

**Fig. 11:**
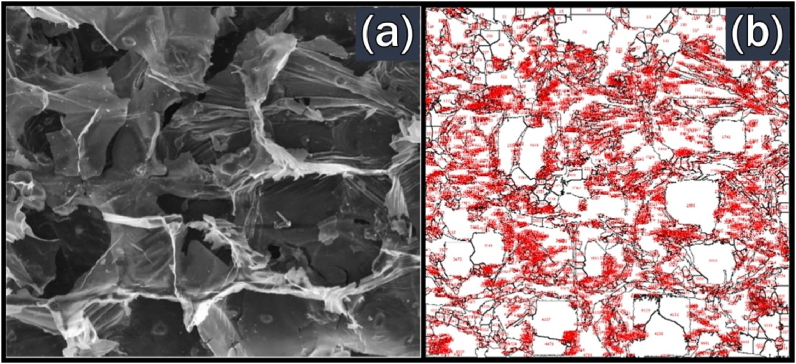
Images of BPS biochar from slow pyrolysis at 500 °C from (a) FESEM; (b) ImageJ analysing no. of pores.

**Fig. 12:**
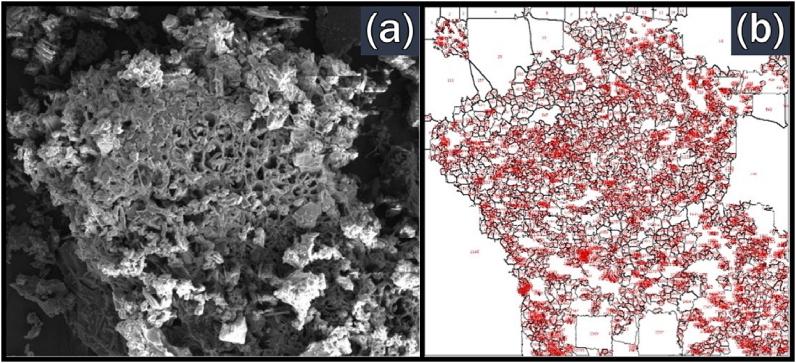
Images of BPS biochar from fast pyrolysis at 500 °C from (a) FESEM; (b) ImageJ analysing no. of pores.

The surface morphology of the BPS biochar obtained from slow and fast pyrolysis is presented in Fig. 11(a) and Fig. 12(a), respectively. These images are the transverse section of the BPS biochar and were magnified by 1000× magnification. This transverse section confirms the multi-cellular structure of BPS as shown by the FESEM image of raw BPS in Fig. 8. Significant differences in surface morphology for BPS biochar could be observed for different types of pyrolysis performed. The cellular structure become irregular for slow pyrolysis of BPS biochar. The pores also can be observed on the surface of biochar. Meanwhile, fast pyrolysis BPS biochar has a defined structure and rough surface.

FESEM images of BPS biochar also were used to estimate the number of pores. It was done by using ImageJ software and the results are shown in Figs. 11(b) and Fig. 12(b). The analysis of ImageJ shows that BPS biochar derived from fast and slow pyrolysis has 2856 and 4722 pores, respectively. The number of pores in fast pyrolysis was less than in slow pyrolysis because heating treatment in fast pyrolysis lasted only less than 2 s. Hence, a smaller number of pores were created in fast pyrolysis than in slow pyrolysis.

The results of FTIR for BPS biochar derived from slow and fast pyrolysis are presented in Table 5 and Table 6, respectively.

**Table 5:** FTIR functional group compositions of BPS biochar derived from the slow pyrolysis process.

Wave number range (cm^−1^)	Wave number (cm^−1^)	Group	Class of compound
3550–3200	3420 2929	O–H bonded	Alcohol and phenols
2400–2100	–	C≡C stretching	Alkyne
1650–1580	1622	C=C stretching	Alkenes
1470–1350	1388	C–H bending	Alkanes
1300–950	1163, 1121, 1009 873, 829, 761, 702, 664, 616	C–O stretching O–H bending	Primary, secondary and tertiary alcohols, phenols, esters, ethers

**Table 6:** FTIR functional group compositions of BPS biochar derived from the fast pyrolysis process.

Wave number range (cm^−1^)	Wave number (cm^−1^)	Group	Class of compound
3550–3200	3409 2968, 2926, 2730	O–H bonded	Alcohol and phenols
2400–2100	2331	C≡C stretching	Alkyne
1650–1580	1622	C=C stretching	Alkenes
1470–1350	1397 1313	C–H bending	Alkanes
1300–950	1161, 1124, 1048, 1006, 981 868, 829, 773, 702, 663, 619, 526	C–O stretching O–H bending	Primary, secondary and tertiary alcohols, phenols, esters, ethers

The result of FTIR analysis in Tables 5 and 6 indicates that the BPS biochar derived from slow and fast pyrolysis at 500 °C has common functional groups. For both BPS biochar, a peak was observed between 3550 and 3200 cm^−1^, associated with –OH groups present in alcohol and phenols. BPS biochar derived from the fast pyrolysis process has additional alkyne in its functional group in the transmittance range of 2400–2100 cm^−1^. Conversely, no triple bond region was detected for BPS biochar obtained from the slow pyrolysis process, informing no C≡C bond in the material.

Meanwhile, a peak approximately at 1622 cm^−1^ was observed in the spectra of both BPS biochar. This peak representing C=C stretching vibration demonstrates the presence of alkene. The peaks at 1388 cm^−1^ for slow pyrolysis BPS biochar and 1397 cm^−1^ and 1313 cm^−1^ for fast pyrolysis BPS biochar are linked with C–H bending, indicating the presence of alkanes. The FTIR results also show the presence of primary, secondary and tertiary alcohols, phenols, esters, and ethers in both BPS biochar with the existence of peaks between 1300 and 950 cm^−1^. The peaks observed in this range are linked with C–O stretching and O–H bending of the BPS biochar. Additionally, the details of the existence and formation of a functional group at the BPS biochar surface derived from both slow and fast pyrolysis may contribute to the information in various applications such as the forecast of biochar recalcitrance against degradation in a soil application.

### 3.4 Limitation of study

This study evaluates the properties of biochar derived from the slow and fast pyrolysis process. However, the characterization of biochar in this study is limited to certain potential applications such as soil amendment and solid biofuel. Further investigations are recommended for the characterization of BPS biochar for other applications such as adsorbents and carbon filler for composites to maximize the potential utilization of BPS biochar. Also, this study only focuses on the biochar derived from the untreated BPS feedstock. Hence, additional pre-treatment on the BPS feedstock is suggested in future studies to improve the properties of BPS biochar obtained from both slow and pyrolysis experiments.

## 4 Conclusion

This study performed slow and fast pyrolysis experiments of BPS feedstock at various terminal temperatures. In both experiments, biochar yield decreased with increasing pyrolysis temperatures. Fast pyrolysis of BPS resulted in a higher percentage yield of biochar (40.3%) than that obtained in the slow pyrolysis experiment (34.9 wt%) at a similar temperature (500 °C) due to the dominant influence of ash during the fast pyrolysis process or the longer residence time experienced by the char in the reactor during the slow pyrolysis process. The high ash content of the BPS feedstock also resulted in a higher biochar yield than the liquid yield obtained from the fast pyrolysis performed at 470–540 °C. Rapid depolymerization due to the higher heating rates during the fast pyrolysis experiment resulted in a lower surface area and porosity of the BPS biochar, as indicated by the BET and FESEM analyses, respectively. The BPS biochar obtained from the slow pyrolysis process has a higher carbon content, heating value, and surface area with lower ash content. Meanwhile, the FTIR analysis showed that the biochar derived at 500 °C of both pyrolysis types has similar functional groups. The characterization of the BPS biochar obtained by slow pyrolysis indicated that the biochar exhibited a higher potential for solid fuel application and soil amendment.

This research also demonstrated the suitability of banana pseudo-stem as a feedstock for the thermochemical conversion process and encouraged higher utilization of BPS, which is one of the residues from banana plantations.

## Author contribution statement

Nurhayati Abdullah: conceived and designed the experiments; performed the experiments; analyzed and interpreted the data.

Rahmad Mohd Taib: performed the experiments; analyzed and interpreted the data; wrote the paper.

Nur Syairah Mohamad Aziz: analyzed and interpreted the data; wrote the paper.

Muhammad Rabie Omar, Nurhafizah Md Disa: contributed reagents, materials, analysis tools or data.

## Funding statement

Nurhayati Abdullah was supported by 10.13039/501100003093Ministry of Higher Education, Malaysia [FRGS/1/2019STG05/USM/02/7], 10.13039/501100004595Universiti Sains Malaysia [1001/PFIZIK/8011113].

## Data availability statement

Data will be made available on request.

Declaration of interest’s statement.

The authors declare no conflict of interest.
